# Machine Learning Accelerated Global Search for Adsorption Geometries of Merocyanine Molecule on Hexagonal Boron Nitride

**DOI:** 10.1002/jcc.70332

**Published:** 2026-02-17

**Authors:** Ritu Tomar, Thomas Bredow

**Affiliations:** ^1^ Mulliken Center for Theoretical Chemistry, Clausius Institute of Physical and Theoretical Chemistry University of Bonn Bonn Germany

**Keywords:** Bayesian optimization, DFT, MACE, merocyanines, ML

## Abstract

The adsorption of the merocyanine dye HB238 on hexagonal boron nitride (hBN) was investigated using a machine learning (ML) assisted global search strategy. A series of MACE machine learning interatomic potentials with higher‐order equivariant message passing were finetuned on density functional theory (DFT) reference data for single and dimer adsorbate configurations, providing accurate surrogate models for the potential energy surface. The Bayesian Optimisation Structure Search (BOSS) was used to search over translational and rotational degrees of freedom of the adsorbed molecules, followed by full PBE/D3 optimisation of the most promising structures. The ML‐accelerated search revealed that HB238 prefers to adsorb in face‐on orientation on hBN surface with the sulfur atoms located near hollow sites; however, the molecule exhibits no strong site selectivity, giving rise to a broad ensemble of configurations within energies 0.1 eV above the global minimum. When two HB238 molecules are adsorbed, they align parallel to each other and lie flat on the surface. Overall, our results demonstrate that combining finetuned ML potentials with Bayesian optimisation enables an efficient and accurate exploration of complex adsorption landscapes and provides fundamental insights into the physisorption of dipolar dyes on 2D insulators. This combined MACE×BOSS approach can be easily extended to investigate organic molecular aggregates on 2D surfaces.

## Graphical abstract

Schematic illustration of HB238 adsorption on hBN(001) explored using Bayesian optimization and a MACE potential. The workflow accelerates potential‐energy‐surface exploration while retaining DFT‐level accuracy for molecular adsorption.

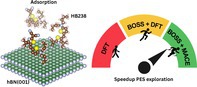

**Abbreviations**
BOSS: Bayesian Optimisation Structure Search
DFT: density functional theory
MACE: higher‐order equivariant message passing potentials
ML: machine learning

## 1 Introduction

Understanding molecular adsorption on solid surfaces is a central problem in catalysis, sensor design, and material functionalization [1, 2, 3, 4]. Two‐dimensional (2D) materials such as hexagonal boron nitride (hBN) and graphene have attracted significant interest due to their exceptional mechanical strength, thermal stability, and wide band gap, making them ideal candidates for various surface chemistry applications [5, 6]. In particular, hBN and graphene provide atomically flat, chemically inert templates on which organic molecules can form highly ordered films with well‐defined epitaxial relationships [7, 8, 9]. Such ordered growth has been reported for rod‐like conjugated molecules such as para‐hexaphenyl and pentacene, which align along high‐symmetry directions of the hBN lattice [9, 10]. However, despite extensive work on related systems, adsorption of merocyanine dyes on hBN has not been studied experimentally or theoretically to our knowledge, leaving an important gap in understanding the templated growth of dipolar chromophores on insulating 2D materials.

The merocyanine dye HB238 (2‐[5‐(5‐dibutylamino‐thiophen‐2‐yl‐methylene)‐4‐*tert*‐butyl‐5H‐thiazol‐2‐ylidene]‐malononitrile) is a prototypical donor‐acceptor chromophore with a large ground‐state dipole moment and tunable optoelectronic properties [11, 12]. On metallic substrates, HB238 has been observed to chemisorb via its sulfur atoms, forming ordered assemblies such as chiral tetramers on Ag(100) [11]. In contrast, adsorption on inert van der Waals surfaces such as hBN(001) is expected to be governed by weaker dispersive and electrostatic interactions, potentially allowing the formation of ordered physisorbed monolayers without strong hybridization effects. Motivated by previous studies of merocyanine and spiropyran derivatives on graphene [13], we investigate here the fundamental adsorption behavior of HB238 on hBN.

Accurately predicting stable adsorption geometries of large and flexible molecules on surfaces is computationally demanding. The potential energy surface (PES) governing molecule‐surface interactions is high‐dimensional and rugged, containing numerous local minima [14]. Traditionally, global optimization and PES sampling methods such as simulated annealing [15], genetic algorithms [16], basin hopping [17], minima hopping [18], metadynamics [19], and Monte Carlo‐minimization [20] have been used to address this challenge, but they are often inefficient for complex organic adsorbates.

Recent advances in machine learning (ML) have revolutionized this domain by providing accurate surrogate models for energy and force prediction at a fraction of the cost of density functional theory (DFT). In particular, ML‐based interatomic potentials such as Message Passing Neural Network (MPNN) architectures have demonstrated DFT‐level accuracy for a variety of systems [21, 22]. Among them, the MACE stands out by incorporating higher‐order equivariant interactions and achieving state‐of‐the‐art performance in capturing short‐range physics in molecules and materials [22, 23]. Recent work has demonstrated that MACE can be effectively finetuned with DFT data to pre‐relax adsorption geometries, discovering new stable configurations while reducing the computational cost by up to 75% compared to brute‐force DFT screening [24].

To efficiently explore the PES using such ML surrogates, Bayesian Optimization (BO) has emerged as a powerful tool for global structure search. The BOSS framework [25, 26, 27] employs Gaussian Process (GP) regression to model the energy landscape and iteratively select new sampling points that balance exploration and exploitation [28]. BOSS has been successfully applied to identify low‐energy adsorption configurations of organic molecules on metallic and 2D surfaces [26].

In this work, we present a hybrid MACE × BOSS framework that combines a MACE‐MP‐0 model [29], fine‐tuned on PBE/D3 data [30, 31], with the BOSS [25] method to efficiently explore the adsorption landscape of the HB238 molecule on hBN. The MACE‐MP‐0 model, originally trained on PBE and PBE+U datasets without dispersion corrections, is finetuned here to include dispersion interactions, which play a crucial role in adsorption phenomena. Within this framework, BOSS performs a five‐dimensional global optimization—two translational and three rotational degrees of freedom—to identify the most stable adsorption geometry. The MACE × BOSS approach achieves a substantial computational speed‐up compared to direct DFT‐based optimizations while retaining near‐DFT accuracy. By combining MACE energy calculation and Bayesian optimization, we overcome the limitations of brute‐force DFT screening, provide the first systematic study of HB238 adsorption on an insulating 2D substrate, and establish a broadly applicable framework for ML‐accelerated exploration of molecule‐surface interactions.

## 2 Computational Methods

### 2.1 Workflow

**FIGURE 1:**
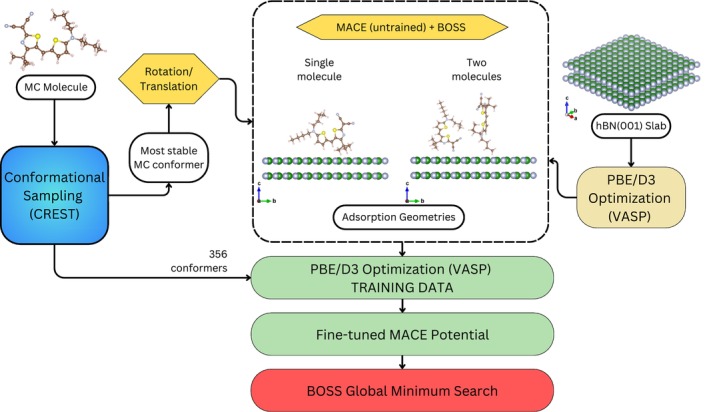
Workflow of the global minimum search of the adsorption structure of HB238 molecule on hBN(001) surface.

The global minimum search for the adsorption structure of the HB238 molecule on the hBN(001) surface is shown in Figure 1. Initially, the conformational landscape of the HB238 molecule was explored using the Conformer‐Rotamer Ensemble Sampling Tool (CREST) [32]. We performed iMTD‐sMTD metadynamics‐based conformer sampling with the GFN2‐xTB [33] semiempirical method as implemented in CREST. After identifying the most stable gas‐phase conformer among the 356 sampled structures, this structure is used as the adsorbate on hBN(001) surface for adsorption structure generation using MACE‐MP‐0 and BOSS. 1191 single‐molecule and 1163 dimer adsorption structures were generated and then optimized with PBE/D3 [30, 31] in VASP [34, 35], creating a comprehensive training dataset. This training dataset generation procedure is implemented as a Python module called “adsgen.” The energy and gradients of the above mentioned PBE/D3 optimized structures were used to train the MACE [22] potential. Finally, the finetuned MACE potential, coupled with BOSS [25], was applied to efficiently perform a global minimum search on the potential energy surface (PES).

### 2.2 Bayesian Optimization Structure Search

BOSS was employed to perform global optimization of HB238 adsorption configurations on the hBN(001) surface using MACE‐trained potentials [25]. For single‐molecule adsorption, a 5‐dimensional configurational space was explored, consisting of x and y lateral translations from 0 to 2.5 Åon the surface and three Euler rotational angles (α, β, γ) of the HB238 molecule. Each angle was sampled uniformly from 0° to 359°. The search was performed using the finetuned potentials as the surrogate model for the potential energy surface. Geometry optimization of each sampled structure was conducted using the PreconLBFGS [36] optimizer in ASE [37] with a force convergence threshold of 0.01 eV/Å. A total of 1000 configurations were evaluated, comprising 20 initial random samples and 980 Bayesian optimization iterations.

The dimer search explored a 10‐dimensional configurational space comprising the combined translations and rotations of two HB238 molecules adsorbed on hBN. Each molecule was allowed two in‐plane translations (x, y) and three Euler rotations (α, β, γ). To ensure comprehensive sampling while avoiding physically unrealistic placements, the translational bounds were derived from extremal positions of two HB238 molecules stacked in various orientations—vertical, side‐by‐side, rotated, diagonal, and overlapping—within the hBN unit cell (see Supporting Information: Figures S1 and S2). From these reference configurations the centre‐of‐mass coordinates were restricted to [4.00,7.50] Å in x and [2.00,7.50] Å in y for Molecule 1, and [4.00,23.50] Å in x and [4.00,20.50] Å in y for Molecule 2. The rotational angles were sampled in discrete $15^{\circ }$ intervals from $0^{\circ }$ to $345^{\circ }$. For each configuration, the predicted α, β, γ values from BOSS were rounded to the nearest multiple of $15^{\circ }$ to reduce the number of redundant configurations and maintain rotational symmetry. Both molecules were initially placed 2.5 Å above the hBN surface, and for vertically stacked configurations the second molecule was offset by an additional 2.5 Å above the first molecule. This vertical spacing eliminated unphysically small distances between the atoms of the two molecules and allowed us to discard implausible structures before optimisation.

### 2.3 MACE Training

Three independent MACE interatomic potentials were refitted to model the PES of HB238 adsorption on the hBN(001) surface. All training structures were optimized at PBE/D3 level prior to MACE model refinement. The first model, referred to as **MACE‐1M**, was re‐fitted on DFT energies and forces of 1191 structures consisting of single HB238 molecules adsorbed on the surface. The second model, **MACE‐1M2M**, used an extended dataset of 2354 structures incorporating both single‐ and two‐molecule adsorption configurations. The third model, **MACE‐1M2M+C**, was trained on a combined dataset of 2660 structures, which included all previous adsorption configurations along with HB238 gas‐phase conformers generated via CREST and optimized at PBE/D3 level. The relative energy of 356 conformers obtained with CREST is shown in Supporting Information: Figure S3.

All models were re‐fitted starting with the “medium” foundation model. The training was performed for a maximum of 2000 epochs with early stopping enabled (patience = 20). The optimizer used was Adam with the AMSGrad variant and a fixed learning rate of 0.001. The loss function was defined as a weighted sum of energy and force terms, with energy and force weights of 10.0 and 1000.0, respectively. Energy reference values were computed using per‐atom averages.

The MACE models were trained with a batch size of four for both training and validation, leveraging CUDA‐enabled GPUs in a distributed setting across four nodes, each equipped with four GPUs. To prevent overfitting, gradient clipping was applied with a threshold of 1.0. Exponential Moving Average (EMA) decay with a rate of 0.995 was employed to smooth parameter updates during backpropagation. The precision for all calculations was maintained at double precision (float64) to ensure numerical stability during gradient updates. Validation was performed after every epoch with a 5% holdout fraction, and the error was measured using Per Atom Mean Absolute Error (PerAtomMAE).

Each of the three refitted MACE models was subsequently integrated into a BOSS to perform global structure optimization over the predicted PES, see previous section.

### 2.4 Density Functional Theory

All density functional theory (DFT) calculations were performed with VASP 6.5.1 [34, 35], employing the Perdew‐Burke‐Ernzerhof (PBE) exchange‐correlation functional within the generalized gradient approximation together with Grimme's D3 dispersion correction [31]. The D3 correction scheme was a suitable choice since the adsorption energies from D4 [38] and VV10 [39] differ from D3 by only 0.003 and 0.330 eV, respectively, while calculation using D3 also has the lowest computational cost as shown in Supporting Information: Table S1. Core‐electron interactions were described using the projector augmented‐wave (PAW) method [40, 41], with PAW‐PBE pseudopotentials from the standard VASP library. A plane‐wave kinetic energy cutoff of 450 eV was applied. Electronic occupancies were determined using Fermi‐Dirac smearing with a width of 0.01 eV. The hBN(001) surface was modeled with a lattice constant of a = 2.51 Å (optimized at the PBE/D3 level) as a periodic slab consisting of two atomic layers. The experimental lattice constant of hBN is 2.50 Å [42]. The convergence calculations for adsorption energy with the size of hBN surface and the number of layers is shown in Supporting Information: Figure S4 and Table S2. For adsorption studies, a 12×12×1 supercell (30.08 Å hBN surface size) was used for single‐molecule adsorption and an 18×18×1 supercell (45.11 Å hBN surface size) for dimer configurations. Given the large surface dimensions, the Brillouin zone was sampled at the Γ‐point only. Ionic relaxations were performed with the conjugate gradient (CG) algorithm, where only the adsorbed HB238 molecule was relaxed while the hBN surface atoms were kept fixed.

## 3 Results and Discussion

### 3.1 MACE Fine‐Tuning

**FIGURE 2:**
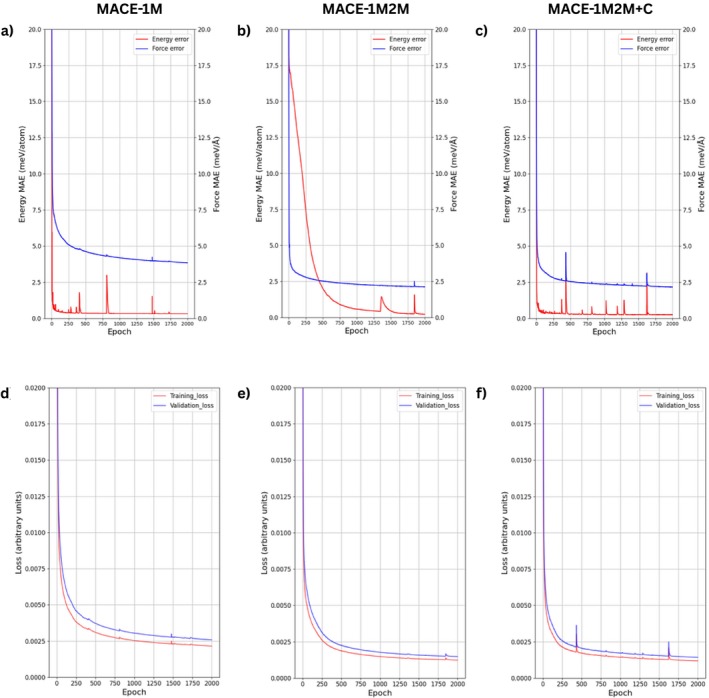
Learning curves for the three refitted MACE potentials developed in this work. Panels (a–c) show the mean absolute error (MAE) of predicted energies in meV/atom (red) and forces in meV/Å (blue) versus training epoch for models trained on single adsorbate (MACE‐1M), single molecule and dimer adsorbate (MACE‐1M2M), and single, dimer and gas‐phase conformer (MACE‐1M2M+C) finetuning datasets, respectively. Panels (d–f) plot the corresponding training (red) and validation (blue) losses versus epoch for each model.

The fine‐tuning performance of the three MACE potentials shown in Figure 2 shows a clear trend of rapid convergence. For the single‐adsorbate model (MACE‐1M) the energy mean absolute error (MAE) drops below 1 meV/atom and the force MAE below 5 meV/Å within the first few hundred epochs, while the validation loss closely tracks the training loss throughout, indicating that the potential fits the single‐molecule adsorption data without overfitting. When the training set is expanded to include two‐molecule configurations (MACE‐1M2M), the initial energy and force errors are larger because the model must learn dimer interactions, but both errors decline steadily and force errors approach those of the single‐molecule model by the end of training and the energy errors drop below 2.5 meV/atom. The most comprehensive model (MACE‐1M2M+C), which also includes gas‐phase conformers, starts with the highest errors but quickly reduces the energy MAE to below 2.5 meV/atom and the force MAE to roughly 2 meV/Å, with a small oscillation around 500 epochs reflecting the assimilation of the conformer data; the training and validation losses remain aligned, demonstrating that the broader dataset does not compromise the model's accuracy.

### 3.2 Global Minima Search for Adsorption Structure With BOSS

To identify the most stable adsorption geometries of the HB238 molecule on the hBN surface we employed the BOSS [25] method, which combined a machine‐learning potential with a Bayesian acquisition function to navigate the high‐dimensional configurational space efficiently. For each configuration returned by BOSS, the energy provided by the fine‐tuned MACE models was used to rank candidate structures, and the energy configurations were then re‐evaluated with DFT to obtain accurate reference energies.

#### 3.2.1 Single‐Molecule Adsorption

**FIGURE 3:**
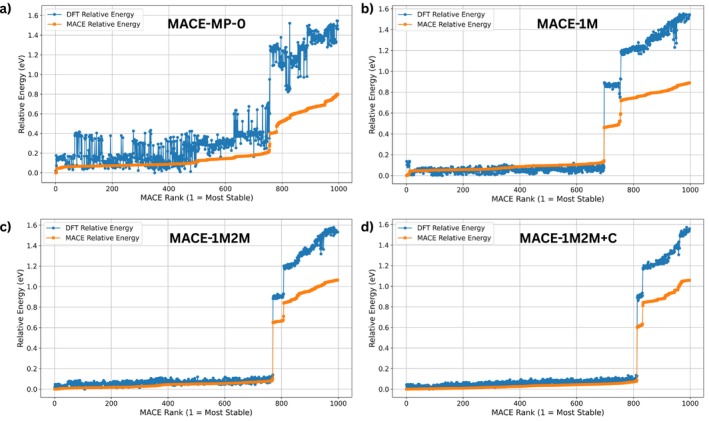
Ranking of relative energies for single‐molecule adsorption obtained with BOSS using the foundation model (a) MACE‐MP‐0 and the fine‐tuned models, (b) MACE‐1M, (c) MACE‐1M2M, and (d) MACE‐1M2M+C. The surrogate energies (orange dots) are compared to respective DFT reference energies (blue dots).

In the single‐adsorbate case, the search space comprised two in‐plane translations and three rotational degrees of freedom as described above. Figure 3 compares the BOSS rankings obtained with the foundation model (MACE‐MP‐0) and the three fine‐tuned models. Each panel plots the DFT relative energy (blue points) and the MACE surrogate potential energy (orange line) as a function of the MACE rank (lower rank corresponds to more stable structures). The PBE/D3 energies shown in Figure 3 are obtained from single point calulations of the structure predicted from MACE × BOSS approach, obtained for the original MACE‐MP‐0 and the three refitted models, respectively. The foundation model shows little correlation between the MACE‐MP‐0 and the PBE/D3 energies: PBE/D3 energies vary widely across the entire rank range and the MACE‐MP‐0 model fails to distinguish low‐energy structures. In contrast, the fine‐tuned models substantially improve the agreement. For the MACE‐1M model, the first 700 ranked structures lie within 0.2 eV of the global minimum, and the finetuned model energy increases smoothly thereafter. This indicates that the model reliably identifies a large family of low‐energy adsorption configurations. The MACE‐1M2M and MACE‐1M2M+C models exhibit similar trends, albeit with slightly smaller deviations between the MACE predicted and PBE/D3 energies at higher ranks.

**FIGURE 4:**
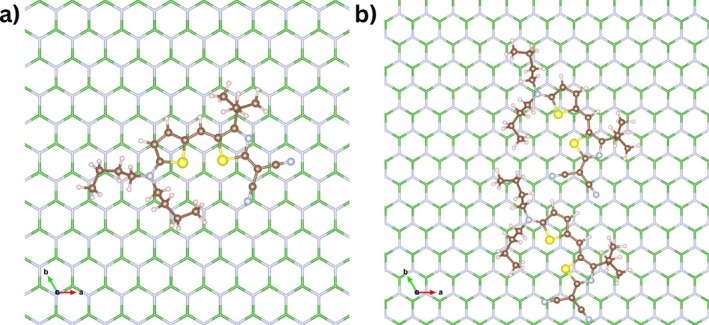
Most stable adsorption motifs from (a) 5D and (b) 10D BOSS search obtained using MACE‐1M2M+C model and relaxed at the PBE/D3 level using DFT. The hBN slab is shown using hexagonal rings in blue and green color and the S, C, N and H atoms are shown using yellow, brown, light blue and light pink color respectively. Only the first layer of the two layer hBN slabs is shown.

**FIGURE 5:**
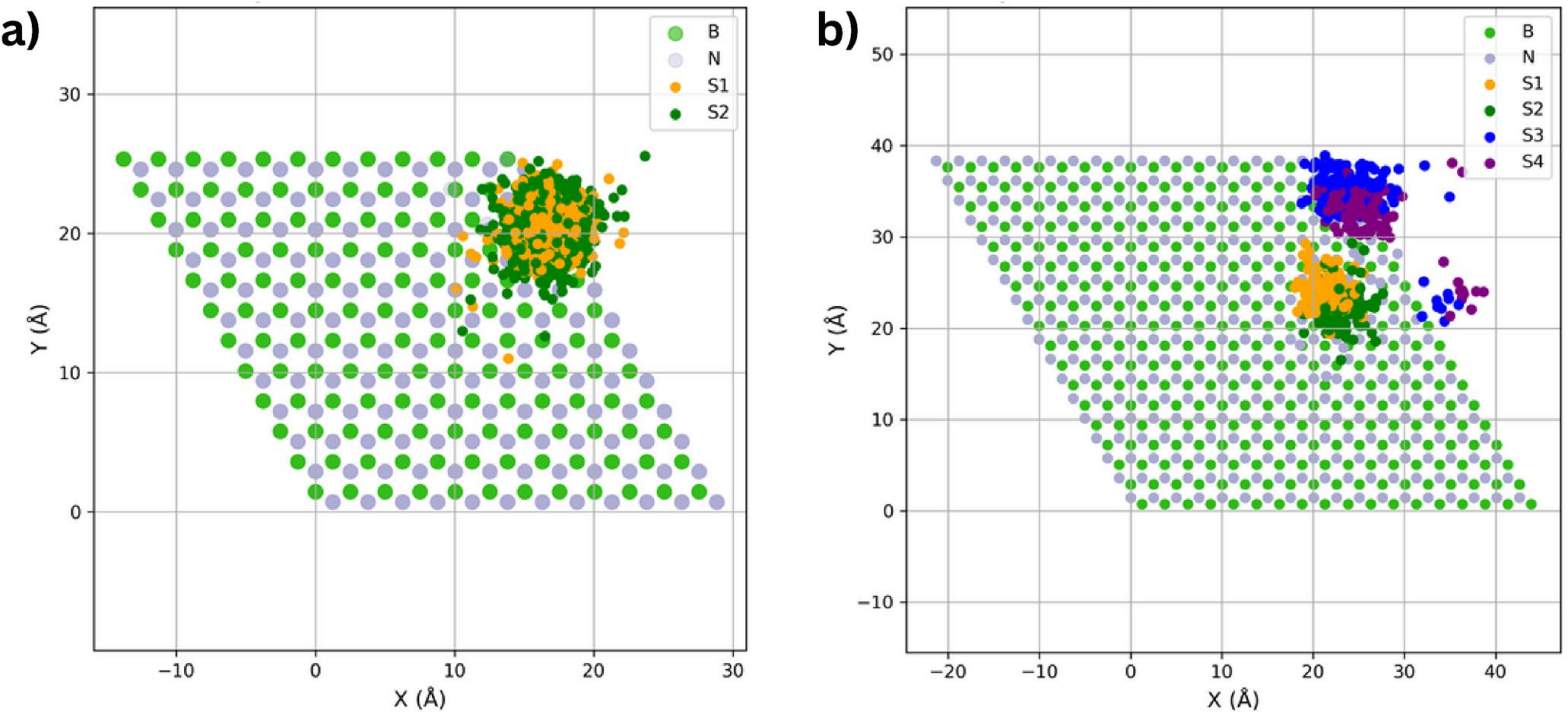
Distribution of sulfur atom positions for all structures with 0.1 eV relative energy of the global minima obtained from the BOSS ensembles with MACE‐1M2M+C. (a) Single‐molecule search: The donor S (orange points) and acceptor atoms (green points), (b) Dimer search: Four clusters (orange, green, blue and purple points) correspond to the four sulfur atoms in the dimer.

Inspection of all structures with 0.2 eV energy difference above the lowest structure obtained with the three finetuned models shows that the HB238 molecule lies flat on the hBN surface. The most stable configuration was obtained using the MACE‐1M2M+C model and is shown in Figure 4. In this configuration, both sulfur atoms are positioned at the hollow sites. The distribution of sulfur positions extracted from all structures within ≤0.1 eV relative energy (773 structures) with respect to the global minimum (Figure 5a) shows the HB238 molecule lacks site‐specific bonding, i.e., the molecule is mobile on the surface. Ten representative monomer adsorption structures within 0.1 eV of the minimum are shown in Supporting Information: Figure S5, with their adsorption energies summarized in Table S3. We also tested the adsorption of HB238 molecule on hBN surface for 300 iteractions using two other MACE potentials, namely MACE‐OMAT‐0 [43] and MACE‐MATPES‐PBE‐0 [44] using MACE × BOSS approach as shown in Figure S6. The MACE‐OMAT‐0 model reliably identifies most of the lower‐energy adsorption structures, however MACE‐MATPES‐PBE‐0 model does not. A further finetuning is required for the latter and can be done using the MACE‐finetuning procedure described above.

#### 3.2.2 Dimer Adsorption

**FIGURE 6:**
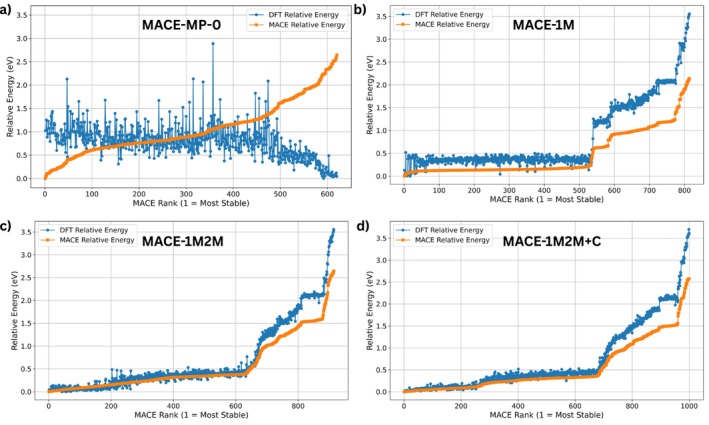
Ranking of relative energies for 10D dimer adsorption obtained with BOSS using the foundation model (a) MACE‐MP‐0 and the fine‐tuned models: (b) MACE‐1M, (c) MACE‐1M2M and (d) MACE‐1M2M+C. The surrogate energies (orange dots) are compared to respective DFT (PBE/D3) reference energies (blue dots) obtained from single‐point calculations of the MACE × BOSS structures.

To study the adsorption of a second molecule as the second step of a deposition process, 10‐dimensional configurational space was explored which included the combined translations and rotations of two HB238 molecules adsorbed on hBN. Figure 6 illustrates the BOSS ranking for this case. The foundation model (MACE‐MP‐0) fails to correlate with the DFT energies and yields a wide spread of predicted low‐energy structures. Fine‐tuning on adsorption data dramatically improves the ranking. The MACE‐1M model captures the low energy plateau but fails to identify the lowest energy structures. From 550 similar energy structures predicted by the model very few are actual minima, this is expected since the model did not contain any dimer structures. The MACE‐1M2M and MACE‐1M2M+C models improve the identification of dimer minima structures. Overall, the surrogate‐guided search locates a rich set of 175 dimer configurations with energies within 0.1 eV of the global minimum.

The lowest‐energy dimer identified by BOSS is shown in Figure 4b. The two HB238 molecules adsorb flat on the surface and align parallel to each other. Ten representative structures within 0.1 eV of the minimum are shown in Supporting Information: Figure S7, with their adsorption energies summarized in Supporting Information: Table S4. The lateral interactions of two molecules on the hBN surface stabilized by intermolecular hydrogen bonding are shown in Figure S8. As in the single‐molecule case, the sulfur positions from the dimer configuration (175 structures within 0.1 eV) also do not show any site preference (Figure 4b), but it is evident that the molecules do not prefer to stack on each other but adsorb flat on the surface, side by side.

## 4 Adsorption Energy

The adsorption energies per molecule were computed as $E_{\text{ads}}=(E_{\mathrm{HB}238/\mathrm{hBN}}-E_{\text{hBN}}-N\;E_{\mathrm{HB}238})$/N, where $E_{\mathrm{HB}238/\mathrm{hBN}}$ is the total DFT energy of the relaxed adsorbate‐slab system, $E_{\text{hBN}}$ is the energy of the bare slab, $E_{\mathrm{HB}238}$ is the energy of an isolated HB238 molecule in the gas phase, and N is the number of adsorbed molecules. A negative value indicates exothermic adsorption.

Table 1 summarises the global minimum adsorption energies for the monomer and dimer configurations. $E_{ads}$ of the most stable monomer adsorption motif is −1.69 eV, while the $E_{ads}$ of most stable dimer configuration is −1.95 eV. The latter is 0.26 eV lower, indicating that lateral interactions between the two molecules provide an additional stabilisation. This extra stabilisation arises from the electrostatic interaction and hydrogen bonding between the cyano group of one molecule and the hydrogen atoms from the pi‐conjugated backbone of the adjacent molecule as shown in Supporting Information: Figure S8. The stronger adsorption of HB238 compared to trans‐azobenzene (−1.49 eV on hBN) [45] is due to the larger molecular size and polarizability of the merocyanine dye. Within the ensemble of 700 low‐energy structures found by BOSS, the adsorption energies vary by less than 0.2 eV, highlighting the flatness of the potential energy surface and suggesting high mobility of the molecule on hBN surface.

**TABLE 1:** Adsorption energy per molecule (eV) for the most stable monomer and dimer adsorption configuration calculated at PBE/D3 level.

Adsorption configuration	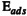 (eV)
Monomer	−1.69
Dimer	−1.95

## 5 Electronic Structure Analysis

**FIGURE 7:**
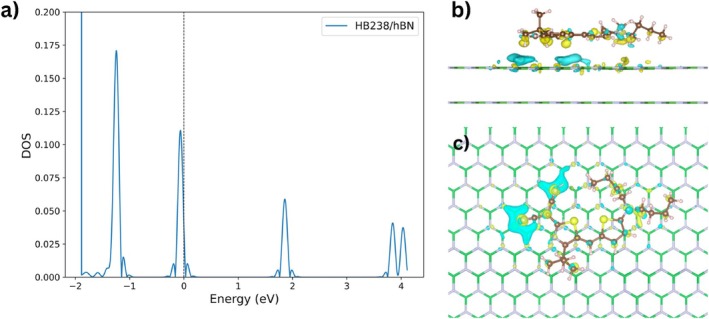
(a) PDOS of HB238 in the most stable adsorption motif on hBN surface, obtained from 5D BOSS search as shown in Figure 4. The black dotted line indicates the Fermi level of the HB238/hBN system. (b) Side view and (c) top view of the charge density difference (CDD) at an isovalue of $5\times 10^{-4}e/{Å}^{3}$, illustrating charge redistribution upon adsorption. Yellow regions correspond to charge accumulation, while cyan regions denote charge depletion.

The electronic interaction between HB238 and the hBN substrate was evaluated through molecular‐projected density of states (PDOS) and charge density difference (CDD) analyses, as presented in Figure 7.

The PDOS projected onto HB238 (Figure 7a) indicates that the frontier orbitals remain sharp and well defined upon adsorption. The HOMO of the HB238 molecule is close to the Fermi level of the combined HB238/hBN system. The persistence of narrow peaks demonstrates that the molecular orbitals retain their localized character with negligible mixing into substrate states, consistent with weak electronic coupling to the hBN lattice.

The CDD maps (Figures 7b and 7c, side and top views) provide spatial insight into charge redistribution at an isovalue of 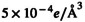. Only minor regions of charge accumulation and depletion are observed around cyano groups of HB238, while the hBN surface remains largely unaffected. This distribution confirms the absence of covalent bonding or significant charge transfer.

## 6 Conclusions

A machine‐learning‐accelerated workflow was developed to explore the high‐dimensional adsorption landscape of the merocyanine dye HB238 on hexagonal boron nitride. MACE interatomic potentials, fine‐tuned on PBE/D3 training data for single and dimer adsorbate configurations, were combined with the BOSS Bayesian optimisation framework to efficiently search the five‐ and ten‐dimensional configurational spaces and identify stable adsorption motifs with chemical accuracy. The machine‐learning surrogate potential enabled the sampling of thousands of candidate structures. The results indicate that HB238 adsorbs flat on hBN surface without strong site specificity, yielding a manifold of monomer and dimer adsorption configurations within 0.2 eV above the global minimum energy. Based on the present theoretical results, it can be predicted that the growth of ordered monolayers of HB238 on hBN is only governed by intermolecular interaction and may be hampered by the lack of directing effects from the surface. This MACE × BOSS workflow is expected to be broadly applicable to other large organic adsorbates and to support the design of hybrid organic‐2D material systems for optoelectronic and sensing applications.

## Funding

This work was supported by Deutsche Forschungsgemeinschaft (Grant No. RTG‐2591).

## Disclosure

The authors gratefully acknowledge funding from the DFG (RTG‐2591 *TIDE Template‐designed Organic Electronics*).

## Conflicts of Interest

The authors declare no conflicts of interest.

## Supporting information




**Data S1:** Supporting Information.

## Data Availability

The data that support the findings of this study are openly available in adsgen at https://github.com/TomarRitu25/adsgen.
