# Supplementary material for: Machine Learning Accelerated Global Search for Adsorption Geometries of Merocyanine Molecule on Hexagonal Boron Nitride
Source: J Comput Chem. 2026 Feb 17;47(6):e70332. doi: 10.1002/jcc.70332 (PMC12911474; doi:10.1002/jcc.70332)
Supplement: Supplementary file 1 — Data S1: Supporting Information. [file JCC-47-0-s001.pdf]

Machine Learning Accelerated Global Search for Adsorption Geometries of Merocyanine molecule on Hexagonal Boron Nitride

Ritu Tomar and Thomas Bredow*

Mulliken Center for Theoretical Chemistry, Clausius Institute of Physical and Theoretical Chemistry, University of Bonn, Beringstraße 4, 53115 Bonn, Germany

E-mail: bredow@thch.uni-bonn.de

Phone: +49 (0)228 733839. Fax: +49 (0)228 739064

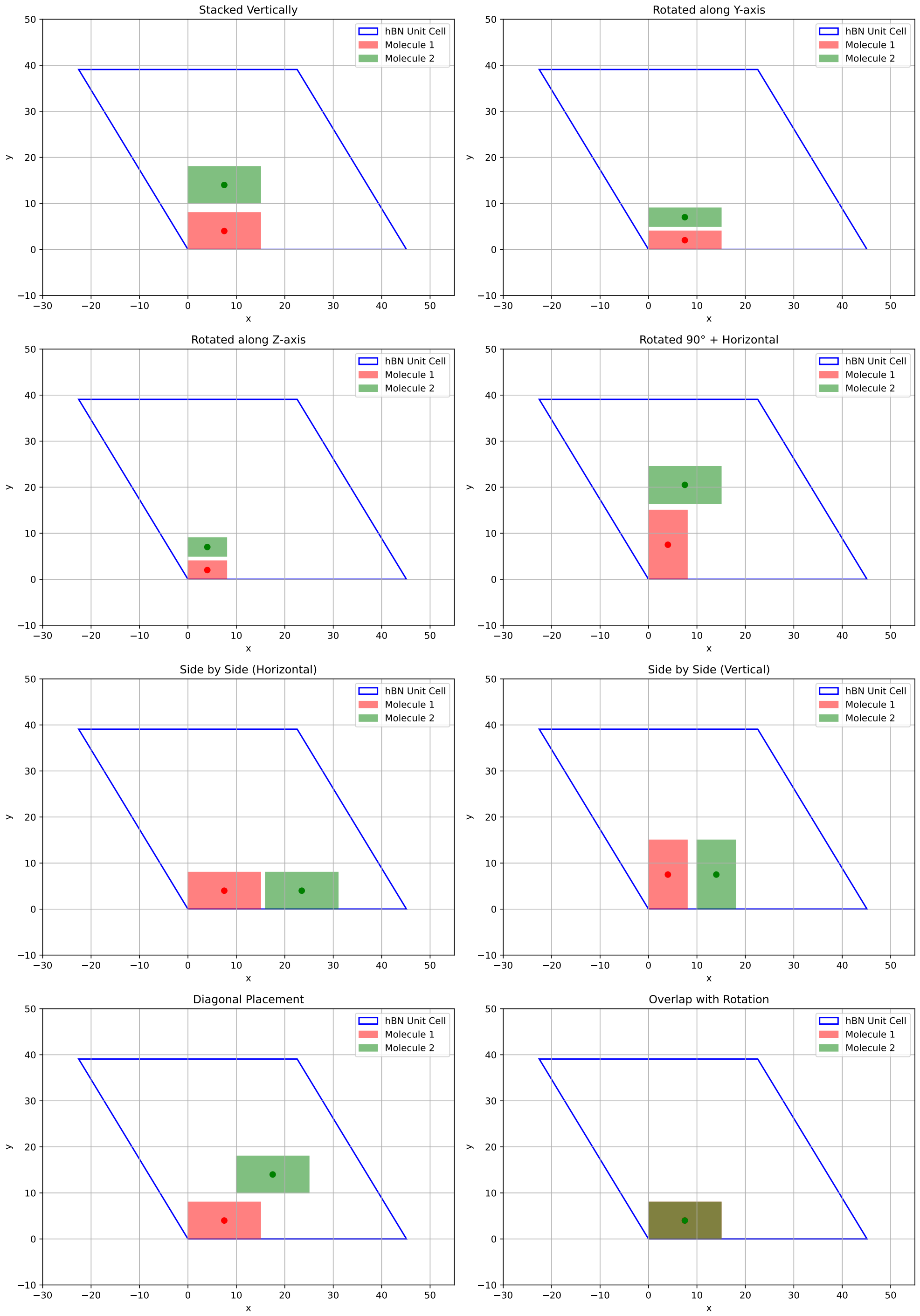

Figure S1: Adsorption configurations of two HB238 molecules on the hBN surface, uses to identify the maximum and minimum x-y center of mass (COM) bounds for BOSS calculation. The eight panels display different configurations—stacked vertically, rotated along the Y- and Z-axes, side-by-side (horizontal and vertical), diagonal, and overlapped with rotation. The blue parallelogram represents the hBN unit cell, with each molecule depicted as a shaded rectangle. Molecule 1 is in red and Molecule 2 in green, with their centers of mass marked by respective colored dots.

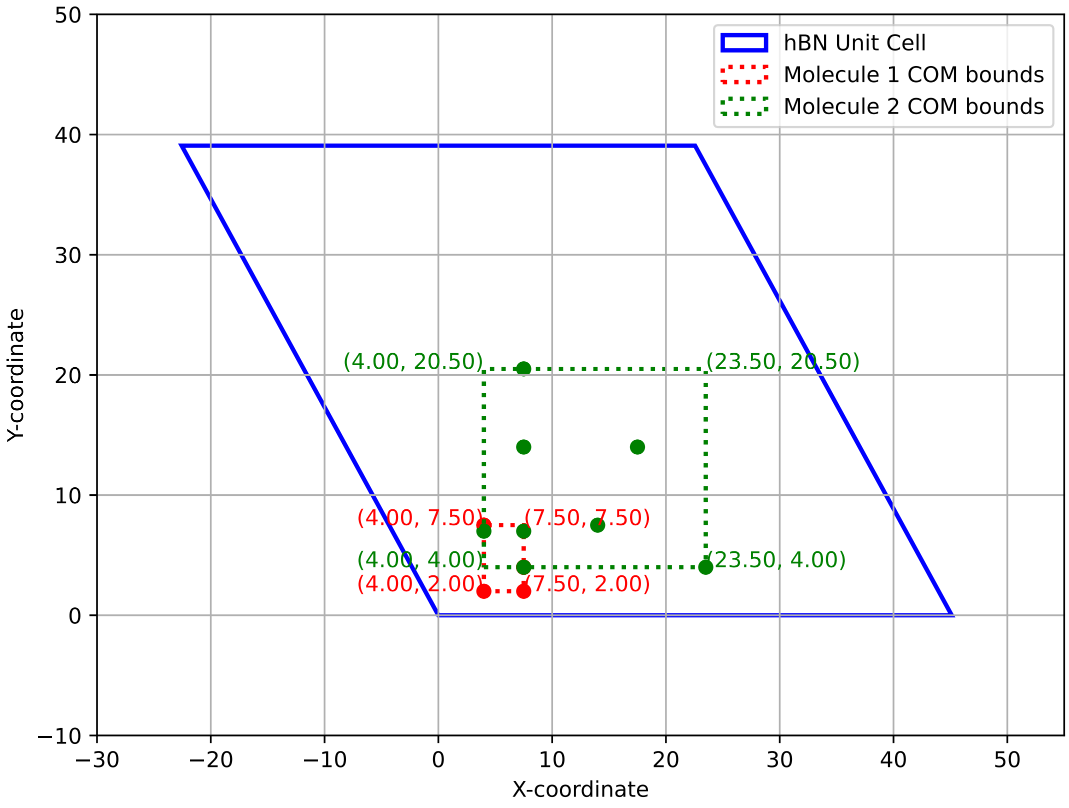

Figure S2: COM bounds of Molecules 1 and 2 on the hBN surface. The plot shows the adsorption of two molecules on the hBN unit cell, represented by the blue parallelogram. Dotted rectangles indicate the bounding boxes around the COM for Molecule 1 (red) and Molecule 2 (green).

Table S1: Comparison of adsorption energies (E_{ads}, in eV) and total computation time (in CPU hours) for the HB238 molecule adsorbed on an Ag surface using different dispersion correction schemes.

Dispersion correction	E_{ads}	Computation Time (hrs)
D3[a]	-1.86	556.63
D4[b]	-1.87	630.15
VV10[c]	-2.19	13141.00
Tkatchenko-Scheffler[d]	-2.78	1129.59

[a]: Reference[1], [b]: Reference[2], [c]: Reference[3], [d]: Reference[4],

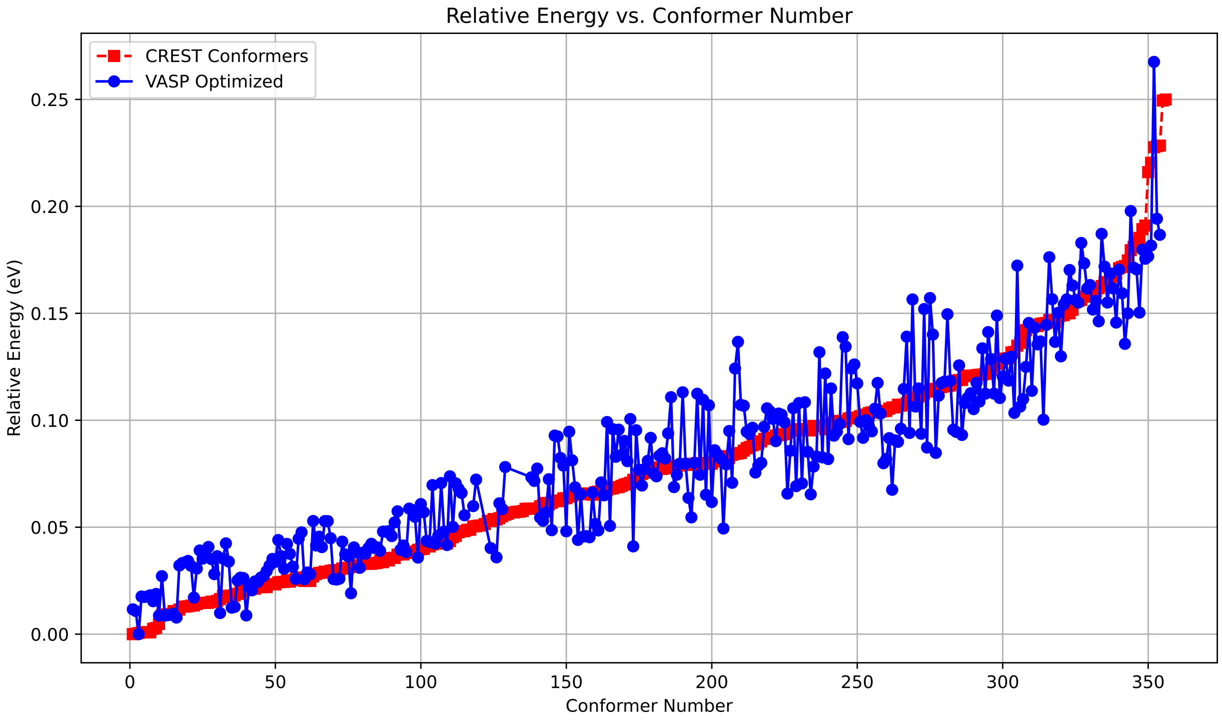

Figure S3: Relative energies of 356 HB238 conformers generated with CREST (red dots) and subsequently re-optimized using VASP at the PBE/D3 level(blue dots).

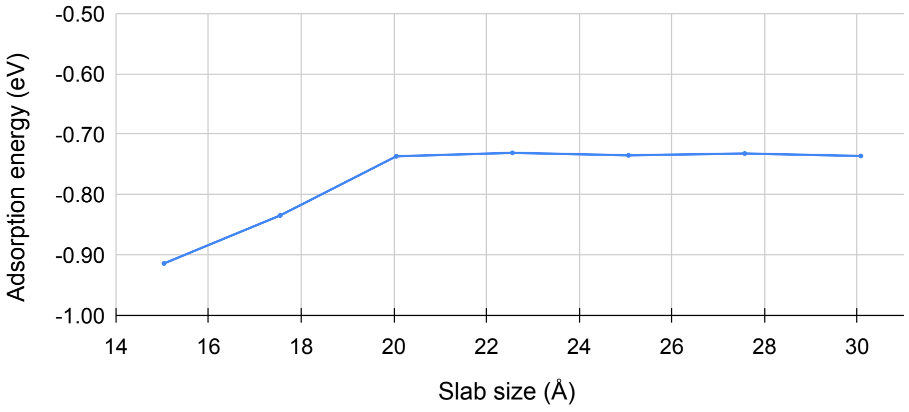

Figure S4: Convergence plot for the adsorption energy of HB238 molecule adsorbed on hBN surface with respect to the hBN slab size (equal expansion along x and y)

Table S2: Adsorption energy of a single HB238 molecule on an hBN surface with varying numbers of hBN layers, calculated using a 12 × 12 × 1 surface supercell.

hBN layer	Adsorption energy
2	-0.85
4	-0.75
6	-0.76

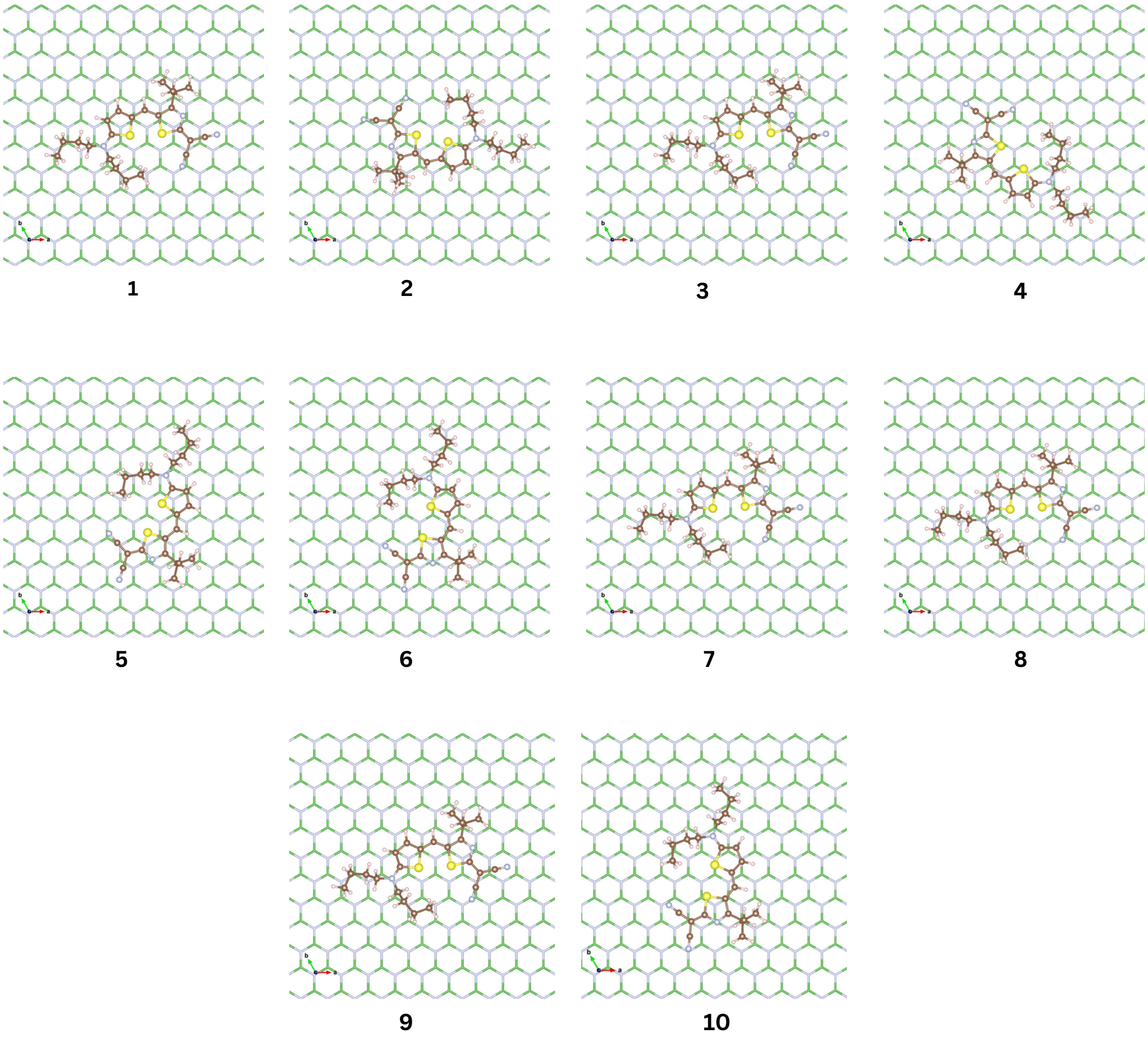

Figure S5: Single HB238 molecules adsorbed on hBN surface arranged in the decreasing order of their stability obtain with BOSSxMACE (MACE-1M2M+C) framework using 5D search and further optimised with PBE/D3.

Table S3: Adsorption energies of single HB238 molecules on the hBN surface, obtained via a 5D configurational search using the BOSS/MACE (MACE-1M2M+C) framework and subsequently optimized at the PBE/D3 level of theory.

Structure	BO step	Adsorption energy (eV)
1	427	-1.69
2	371	-1.68
3	253	-1.67
4	497	-1.66
5	704	-1.66
6	921	-1.65
7	410	-1.60
8	882	-1.60
9	395	-1.58
10	366	-1.58

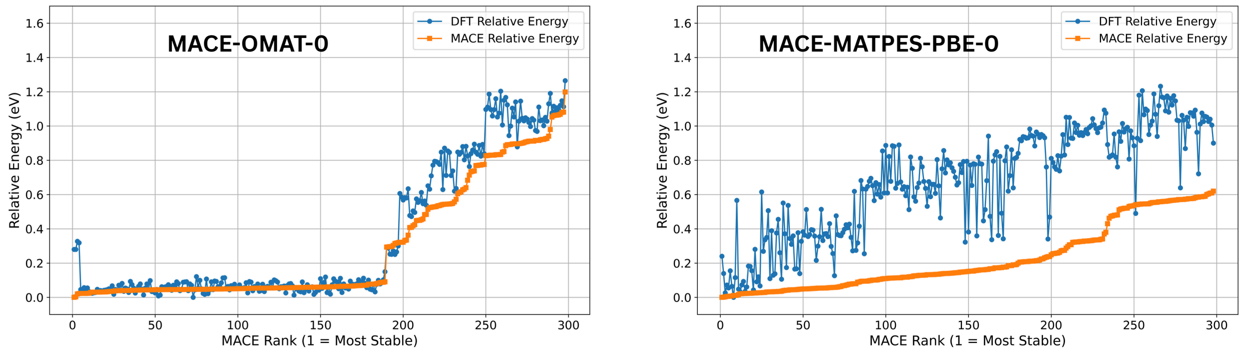

Figure S6: Relative energy rankings for single-molecule configurations generated by BOSS using the MACE-OMAT-0 (left) and MACE-MATPES-PBE-0 (right) foundation models with D3 dispersion corrections. Predicted energies from the surrogate models (orange dots) are shown alongside the corresponding DFT reference values calculated as single point energies of the same adsorption configuraton using PBE/D3 (blue dots).

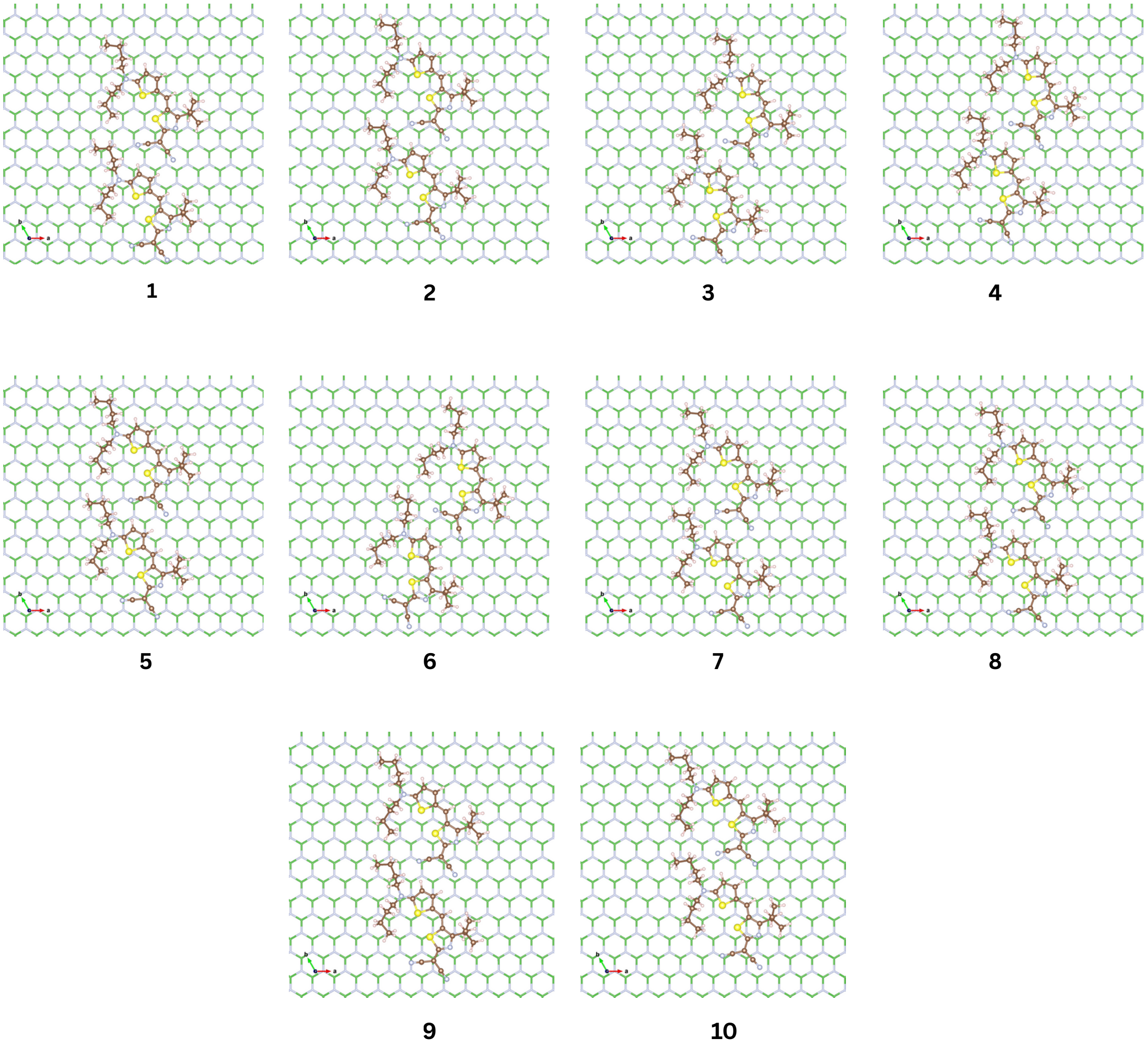

Figure S7: Two HB238 molecules adsorbed on hBN surface arranged in the decresing order of their stability obtained with BOSSxMACE (MACE-1M2M+C) framework using 10D search and further optimised with PBE/D3.

Table S4: Adsorption energies of two HB238 molecules on the hBN surface, obtained via a 10D configurational search using the BOSS/MACE (MACE-1M2M+C) framework and subsequently optimized at the PBE/D3 level of theory.

Structure	BO step	Adsorption energy (eV)
1	298	-1.95
2	931	-1.93
3	480	-1.93
4	604	-1.92
5	924	-1.90
6	782	-1.90
7	335	-1.89
8	539	-1.87
9	762	-1.86
10	693	-1.84

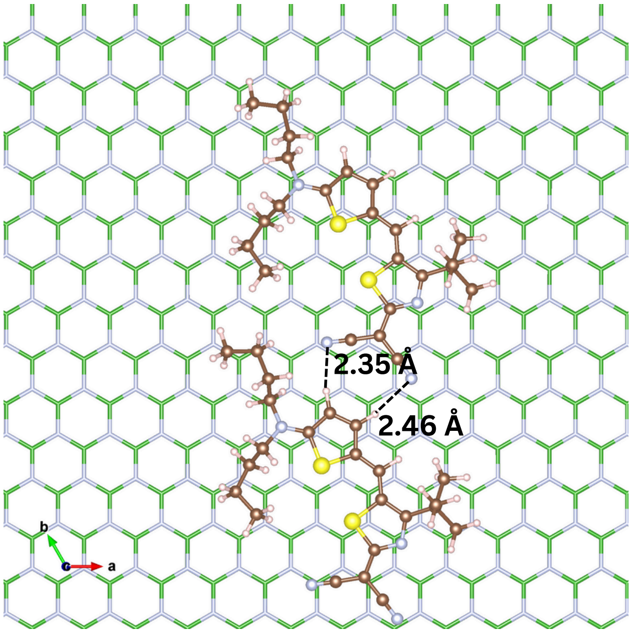

Figure S8: Illustration of intermolecular H-bonding interactions of two HB238 molecules adsorbed on hBN surface.

References

(1) Grimme, S.; Antony, J.; Ehrlich, S.; Krieg, H. A consistent and accurate ab initio parametrization of density functional dispersion correction (DFT-D) for the 94 elements H-Pu. *J. Chem. Phys* **2010**, *132*.

(2) Caldeweyher, E.; Bannwarth, C.; Grimme, S. Extension of the D3 dispersion coefficient model. *J. Chem. Phys.* **2017**, *147*, 34112.

(3) Sabatini, R.; Gorni, T.; De Gironcoli, S. Nonlocal van der Waals density functional made simple and efficient. *Phys. Rev. B* **2013**, *87*, 041108.

(4) Tkatchenko, A.; Scheffler, M. Accurate Molecular Van Der Waals Interactions from Ground-State Electron Density and Free-Atom Reference Data. *Phys. Rev. Lett.* **2009**, *102*, 073005.
